# Influence of Prednisolone and Alendronate on the *de novo* Mineralization of Zebrafish Caudal Fin

**DOI:** 10.1002/jbm4.10435

**Published:** 2020-12-05

**Authors:** Fabio Rocha Bohns, Yann‐Rong Shih, Yung‐Jen Chuang, Riaz Akhtar, Po‐Yu Chen

**Affiliations:** ^1^ Department of Materials Science and Engineering National Tsing Hua University Hsinchu Taiwan; ^2^ Department of Mechanical, Materials and Aerospace Engineering University of Liverpool Liverpool UK; ^3^ International Intercollegiate Ph.D. Program National Tsing Hua University 101 Hsinchu Taiwan; ^4^ Department of Medical Science and Institute of Bioinformatics and Structural Biology National Tsing Hua University Hsinchu Taiwan

**Keywords:** INDENTATION (NANO/MICRO), ANALYSIS/QUANTITATION OF BONE, OSTEOPOROSIS, DISEASES AND DISORDERS OF/RELATED TO BONE, BIOMECHANICS, ORTHOPAEDICS, OTHER, THERAPEUTICS, MATRIX MINERALIZATION, BONE MATRIX

## Abstract

Dysregulated balance between bone resorption and formation mediates the onset and progression of osteoporosis. The administration of prednisolone is known to induce osteoporosis, whereas alendronate is commonly used to reverse the process. However, the assessment of the effects of such medicines on the nanostructure of bone remodeling and mechanical properties remains a major technical challenge. The aim of this study was to apply various analytical approaches to evaluate the compositional, morphological, and mechanical properties of regenerative zebrafish caudal fin bony rays affected by prednisolone and alendronate. Adult wild‐type AB strain zebrafish were first exposed to 125μM of prednisolone for 14 days to develop glucocorticoid‐induced osteoporosis. Fish fins were then amputated and let to regenerate for 21 days in tank water containing 30μM of alendronate or no alendronate. The lepidotrichia in the proximal and distal regions were evaluated separately using confocal microscope, scanning electron microscope, electron‐dispersive spectroscopy, Raman spectroscopy, atomic force microscopy, and a triboindenter. As expected, prednisolone led to significant osteoporotic phenotypes. A decrease of Ca/P ratio was observed in the osteoporotic subjects (1.46 ± 0.04) as compared to the controls (1.74 ± 0.10). Subsequent treatment of alendronate overmineralized the bony rays during regeneration. Enhanced phosphate deposition was detected in the proximal part with alendronate treatment. Moreover, prednisolone attenuated the reduced elastic modulus and hardness levels (5.60 ± 5.04 GPa and 0.12 ± 0.17 GPa, respectively), whereas alendronate recovered them to the pre‐amputation condition (8.68 ± 8.74 GPa and 0.34 ± 0.47 GPa, respectively). As an emerging model of osteoporosis, regrowth of zebrafish caudal fin was shown to be a reliable assay system to investigate the various effects of medicines in the *de novo* mineralization process. © 2020 The Authors. *JBMR Plus* published by Wiley Periodicals LLC on behalf of American Society for Bone and Mineral Research.

## Introduction

Osteoporosis is a chronic degenerative disease of bones, characterized by the imbalance between bone resorption by osteoclasts and the ossification process by osteoblasts during the remodeling process.^(^ ^1^ ^)^ The increased bone remodeling activity in favor of osteoclasts often leads to reduced bone‐mineral density (BMD), high porosity and, consequently, in the deterioration of mechanical properties and high fracture risk.^(^ ^1^, ^2^, ^3^ ^)^ As people's life expectancy increases,^(^ ^4^ ^)^ osteoporosis may cause a significant impact on the quality of life of elderly people and burden the world's economy in a short time.^(^ ^2^, ^5^ ^)^

Prednisolone (PN) is a glucocorticoid often used as a treatment to inflammation and its prolonged administration is well known to cause secondary osteoporosis in humans.^(^ ^6^ ^)^ In order to counteract the adverse effects of the continuous use of glucocorticoids, antiresorptive agents (eg, bisphosphonates) has been suggested.^(^ ^7^, ^8^, ^9^ ^)^ Among the available bisphosphonates, alendronate (ALN) is a compound that could regulate bone remodeling by promoting osteoclasts apoptosis and osteoblast differentiation.^(^ ^7^, ^10^ ^)^ The long‐term administration of ALN has been shown to increase continuously BMD of the lumbar spine, with no serious adverse effects related to fractures, reversing the osteoporotic phenotype in postmenopausal women.^(^ ^11^ ^)^ Despite the positive outcomes, little is known of how mineralized tissues, specifically for human diseased bones, are influenced by these anti‐mineralogenic and pro‐mineralogenic medicines at the multiscale level. The evaluation of the compositional, morphological, and mechanical characteristics is particularly limited.^(^ ^12^ ^)^ The use of animal models has therefore been proposed.^(^ ^13^, ^14^ ^)^

Zebrafish are small teleost fish and have emerged as a suitable model to study anti‐mineralogenic^(^ ^15^, ^16^, ^17^, ^18^ ^)^ and pro‐mineralogenic^(^ ^7^, ^19^ ^)^ compounds due to their feasibility for in vivo imaging, easy follow‐up potential, and their conserved bone physiology with humans.^(^ ^15^, ^20^, ^21^, ^22^ ^)^ One of the most notorious characteristics of zebrafish is their ability to regenerate fins after resection and repair minor injuries in their skull.^(^ ^20^, ^23^, ^24^, ^25^ ^)^ Their caudal fin is composed of ~18 rays of intramembranous dermal bones (lepidotrichia) that, after resection, triggers a process to be repaired, attaining similar shape and function as the inborn appendage.^(^ ^20^, ^24^, ^25^, ^26^, ^27^, ^28^ ^)^ It has been demonstrated that dedifferentiated osteoblasts play a key role in lepidotrichia's regenerative process.^(^ ^27^ ^)^ Conversely, the role of osteoclasts in the fin's regeneration process is still not clear.^(^ ^23^ ^)^ However, when PN is administered simultaneously to a regenerative process, the recruitment of these cells to the injured site is irregular,^(^ ^23^, ^27^, ^29^ ^)^ which may impair the regrowth and properties of the newly formed fin. A commonly used approach to assess the changes caused by osteoactive compounds on zebrafish is by using a fluorescent dye (eg, Alizarin red‐S [AR‐S]) to stain their mineralized tissues.^(^ ^2^ ^)^ The mineral depletion caused by bone diseases (eg, glucocorticoid‐induced osteoporosis), has been demonstrated to decrease the detected fluorescence signal intensity.^(^ ^15^ ^)^ On the other hand, increased intensities were observed when bisphosphonate compounds (eg, ALN) were administered.^(^ ^19^ ^)^

The mineralogenic effects of PN and ALN on the zebrafish skeleton are not novel^(^ ^2^, ^15^, ^17^, ^19^ ^)^; however, little is known on their aftereffects in the multiscale level. Thus, the aim of this study is to evaluate the appearance, composition, and mechanical properties of zebrafish caudal fin bones, before and after resection, affected, or not, by PN or ALN. The application of resection/regeneration processes enables the longitudinal evaluation of the same individual, which may minimize any potential effects caused by each individual characteristic (eg, genetic variation) and also complies with the three R's in the ethical guidelines for animal research (ie, reduction, replacement, and refinement).^(^ ^22^, ^30^ ^)^ Understanding how medicines influence the properties of caudal fin bony rays in zebrafish could further the understanding of the pathophysiological of diseased mineralized tissues. The null hypothesis to be tested is that the different medicine treatments do not affect the properties of zebrafish caudal fin bones.

## Materials and Methods

### Zebrafish maintenance

Adult 3‐month‐old wild‐type AB strain zebrafish (*n* = 46), with average length of 30.73 ± 4.32 mm, were obtained from the Taiwan Zebrafish Core Facility at Academia Sinica (TZCAS). The fish were maintained inside tanks in recirculating water system under a 14/10 hour light/dark (l/d) cycle at 28°C and were fed twice a day, as described in the Zebrafish Book,^(^ ^31^ ^)^ until they were sorted to experimentation. The experimental use of zebrafish was approved by the Experimental Animal Care and Use Committee of National Tsing Hua University (NTHU) (IACUC approval number: 10048). The steps used to evaluate the zebrafish caudal fins in this work are represented in Supplementary Fig. S1.

### Lepidotrichia imaging and glucocorticoid‐induced osteoporosis model

Thirty‐one fish (*n* = 31) were randomly selected from the initial 46 and were immersed for 15 min in a 0.01% AR‐S solution (Sigma‐Aldrich, St. Louis, MO, USA) with its pH adjusted to ~7.4 with KOH solution. The fish were rinsed in fish tank water for three times every 5 min to remove the excess of AR‐S staining.^(^ ^21^ ^)^ Then, the fish were anesthetized individually in a 200‐mL solution containing 70 ppm of Tricaine (MS‐222; Sigma‐Aldrich), adjusted to pH ~7.2 (± 0.1) with sodium hydroxide (NaOH), 130 μL of isoflurane (Alfa Aesar, Loughborough, Leicestershire, UK) and ethanol (isoflurane:ethanol = 1:9), as described.^(^ ^32^ ^)^ The anesthetized fish were placed carefully onto a cell and tissue culture disk (Biofil; Kaohsiung, Taiwan) and the tiles images of the bright field (T‐PMT–T3) and fluorescent light (Cy3‐T3) of the caudal fin bony rays were taken with an inverted confocal microscope (LSM 800; Carl Zeiss, Oberkochen, Germany). The pinhole size was set to 4.49 A.U., with a bit depth of 8 bits and the detection gain were set to 170 V and 680 V (T‐PMT‐T3 and Cy3‐T3, respectively). The pictures obtained were labeled as zero days (0 day). The other 15 fish were raised separately, in fish tank water only, for later use.

After being imaged, the zebrafish were put individually into 120 mL of fish tank water containing PN (Sigma‐Aldrich) previously dissolved in 0.1% of dimethyl sulfoxide (DMSO; J.T. Baker, Phillipsburg, NJ, USA), in a final concentration of 125μM. For 14 days, 1/3 of water with the medicine was changed daily. After 14 days (14 d), the fish were AR‐S stained, anesthetized and analyzed again by an inverted microscope using the same parameters as described previously. The caudal fins images had the pixel intensity of the ventral lobe bony rays measured with ImageJ 1.52a software (NIH, Bethesda, MD, USA; https://imagej.nih.gov/ij/)). The pixel intensities were then transformed into percentage and both groups of images were compared. Five fish died naturally during the treatment.

### First fin resection

The fish not previously exposed to any medicines (*n* = 15) were sorted and put individually into containers with fish tank water only. Individually, both the untreated and fish with osteoporotic phenotype (*n* = 26) were anesthetized and the caudal fin resection were performed approximately three segments proximal to the first lepidotrichia bifurcation. The fins amputated from the group of untreated fish were labeled as the control (CTRL); the fins from the group with osteoporosis were labeled as GIOP. The fish were put into a recovery basin containing fish tank water only before the treatment started.

### Medicine administration and *de novo* mineralization

The amputated living fish were put into numbered containers, and were assigned into three groups according to the treatment proposed. The fish with osteoporosis were divided into two treatment groups: 13 fish (*n* = 13) were assigned to tanks filled with 120 mL of fish tank water only, while the other half (*n* = 13) were added to tanks with 120 mL water containing ALN in a concentration of 30μM. The untreated fish (*n* = 15) were put to regenerate in tanks containing 120 mL of fish tank water only. The zebrafish remained in numbered containers for 21 days, and 1/3 of the water with and without medicine were refreshed daily. The regeneration process for each fish was followed up, and images were recorded at 1, 4, 7, 11, 14, and 21 days postamputation (dpa). In each of the time points, AR‐S staining, water rinsing, and anesthetic procedure were performed individually in each fish; the images were obtained with an inverted confocal microscope, as described previously in the “Lepidotrichia imaging and glucocorticoid‐induced osteoporosis model” section. Before 1 dpa analysis was performed, seven fish (*n* = 7) with osteoporotic phenotype died naturally (three from the group assigned to fish tank water only and four fish from the ALN intervention group).

The caudal fin bony rays *de novo* mineralization was quantified in accordance to the parameters suggested by Cardeira and colleagues.^(^ ^20^ ^)^ A summary of these parameters is shown in Table 1*A*. Using ImageJ, the values extracted from the images analyzed were transformed into distance or area (mm or mm^2^, respectively) by calibrating the pixel values with the scale bar shown in the images, before the calculations were performed.

**Table 1:** Summary of the Mineralization/Regeneration Parameters Proposed by Cardeira and colleagues^(^ ^20^ ^)^ to Explore (*A*) the Mineralogenic Performance in Zebrafish Caudal Fin Models and (*B*) Parameters Used to Obtain the CTRL and Experimental Groups Proposed

(*A*) *de novo* mineralization quantification parameters obtained by ImageJ^(^ ^20^ ^)^		
RMA: the real mineralized area was determined by the area inside the full contour of the mineralized rays, from the resection plane to the most distal bony rays’ tips		
RAY: the mean ray width was defined as de mean value extracted from the length of each fin ray, measured using the first segment below the resection extension		
REG: the total regenerated area was obtained from the full contour of the caudal fin tissue		
STU: the stump width corresponded to the length of the amputation plane		
RMA/RAY: Mineral deposition within the regenerated lepidotrichia		
REG/STU: Regenerated tissue within the resection plane length		

(*B*) Zebrafish treatment condition		
Group	Resection	Condition
CTRL	1st	Untreated zebrafish
GIOP	1st	Zebrafish treated with 125μM PN for 14 days
CTRL_REGEN_	2nd	CTRL condition + fish tank water only for 21 days
GIOP_REGEN_	2nd	GIOP condition + fish tank water only for 21 days
ALN_REGEN_	2nd	GIOP condition + fish tank water +30μM ALN for 21 days

### Second fin resection

After 21 dpa, the regenerated fins were resected using procedures as described previously in the “First fin resection” section of this text. The fins extracted from the fish immersed in fish tank water only (no previous disease condition) were labeled as CTRL_REGEN_; the caudal fins amputated from the group of fish that previously developed osteoporotic‐like bones, and were let to regenerate in fish tank water only, were called GIOP_REGEN_ and, finally, the fish with osteoporosis condition treated with ALN were labeled as ALN_REGEN_. A summary of the treatment groups proposed are shown in Table 1*B*.

### Mineral morphology and content

The amputated zebrafish caudal fins (*n* = 3; each group) were put onto microscope glass slides and were bleached with 5% sodium hypochlorite solution (NaOCl; J.T. Baker) in order to remove external tissue and fatty components.^(^ ^33^ ^)^ The bleaching solution was washed out with deionized water and the fins were dehydrated in ethanol series (50%, 75%, and 100%) for 15 min sequentially. The exposed bones were then separated into proximal and distal regions by performing a section approximately two segments proximal to the cleft. The proximal and distal parts were rapidly frozen under liquid nitrogen and crushed into small particles with mortar and pestle. The still frozen particles were critical‐point‐dried (CPD) in a Samdri‐795 critical point dryer (Tousimis, Rockville, MD, USA) and evaluated. The morphology of proximal and distal regions of the fins were observed using a Scanning Electron Microscope (SEM) SU8010 (Hitachi, Chiyoda, Japan) equipped with an Energy‐Dispersive X‐ray Spectroscope (EDS). The Calcium (Ca) and Phosphorus (P) atomic percentages, were recorded in five different sites using EDS. The areas of analysis were fixed to 7 × 7 μm^2^ and the equipment voltage was set to 15 kV for both characterization methods.

### Raman spectroscopy

Raman spectra were obtained with a Raman spectrometer iHR550 (Horiba Scientific Ltd., Kyoto, Japan) equipped with a laser confocal microscope IX71 (Olympus, Tokyo, Japan). A magnification ×10 lens and a laser source with wavelength of 632.81 nm were used and the equipment acquisition time was set to 20 s with three co‐additions. One amputated caudal fin (*n* = 1), with similar size and with 18 lepidotrichia each were selected and transferred to opaque 3D printed polymer substrates. The bones were exposed with NaOCl and subsequently dehydrated in ethanol series. A total of 96 equidistant points (~700 μm distant) had the spectrum recorded and the area under the phosphate peak (~960 cm^−1^) were calculated using LabSpec 6 software (Horiba Scientific Ltd.). The integrated values were plot as a heat color map relatively to the position analyzed.

### Cross‐section procedure

Two amputated fins of each group (*n* = 2) were gently placed into a cryomold (TissueTek®; Sakura Finetek Europe B.V., Alphen aan den Rijn, the Netherlands) and were totally covered with Optimal Cutting Temperature (OCT) compound (TissueTek®). The compounds were indirectly frozen using liquid nitrogen, covered with aluminum foil and stored at −21°C until used. The blocks were cut into 30‐μm‐thick slices, perpendicular to the bony rays' direction of growth, at −21°C, with a microtome CM3050 S (Leica Microsystems GmbH, Wetzlar, Germany), as described.^(^ ^34^ ^)^ The used thickness is over the minimum standard accepted to submicron analyses.^(^ ^35^ ^)^ The sliced samples were fixed in properly labeled microscope glass slides and the excess of OCT was washed using phosphate‐buffered solution (PBS) with pH of ~7.4, formulated as described by Chazotte.^(^ ^36^ ^)^ One of the cross‐sectioned samples was used in the surface roughness analysis, whereas the other was used for mechanical evaluation.

### Surface profile and roughness

The surface profiles of sliced samples were observed using an ICON® Atomic Force Microscope (AFM; Bruker Optics Inc., Billerica, MA, USA). The AFM was operated in tapping mode in air using a nonconductive silicon nitride (Si_3_N_4_) tip with a nominal radius of 4 μm (Nanosensors, Neuchâtel, Switzerland). The spring constant was 42 N/m with resonant frequency of 330 kHz. The equipment was set to a scan rate of 0.99 Hz and the images were captured with a resolution of 256 pixels/line. The third lepidotrichia from dorsal part was used in this technique. One slice between the second and third segment proximal to resection plane, and other between the second and third segment near to the distal tips, had the surface profile and roughness recorded. Five areas of 2 μm^2^ were measured and the average value of Ra was calculated with the software NanoScope Analysis v1.4 (Bruker).

### Mechanical evaluation

Three consecutive slices from each proximal and distal region had their mechanical properties evaluated. A Tribo‐Indenter (TI 980; Bruker) equipped with a Berkovich tip was used in order to measure the reduced elastic modulus (*E*_*r*_) and hardness (*H*) of the samples. The depth of each indentation was controlled to 100 nm and the loading, holding, and unloading times were set to 5 s each. A series of ~26 and ~20 indentations, for proximal and distal sites, respectively, distributed in three lines, were performed at the central point of the hemi rays of the third dorsal lepidotrichia. The distance between each measurement was set to 100 nm in order to minimize the effects of neighbor indentations. The load versus displacement curves were plotted and the curves presenting anomalies (eg, shoulder) were removed from the final results. In addition, both *E*_*r*_ and *H* were calculated in relation to their mineral ratios obtained from the proximal regions.

### Statistical analysis

Descriptive analysis was performed on SEM, Raman mapping, and AFM images. All the statistical analyses were performed with SigmaPlot 12.0 (Systat Software, San Jose, CA, USA). The normality of the samples was verified using Shapiro‐Wilk test (*p* < .01). The *t* test was used to verify the statistical significance of glucocorticoid‐induced osteoporosis treatment (*p* < .01). Two‐way ANOVA was used for the remaining pixel‐related measurements, elemental analyses, surface roughness, *E*_*r*_, and *H* (*p* < .01).

## Results

### Effects on mineralization by fluorescent imaging

**Fig 1:**
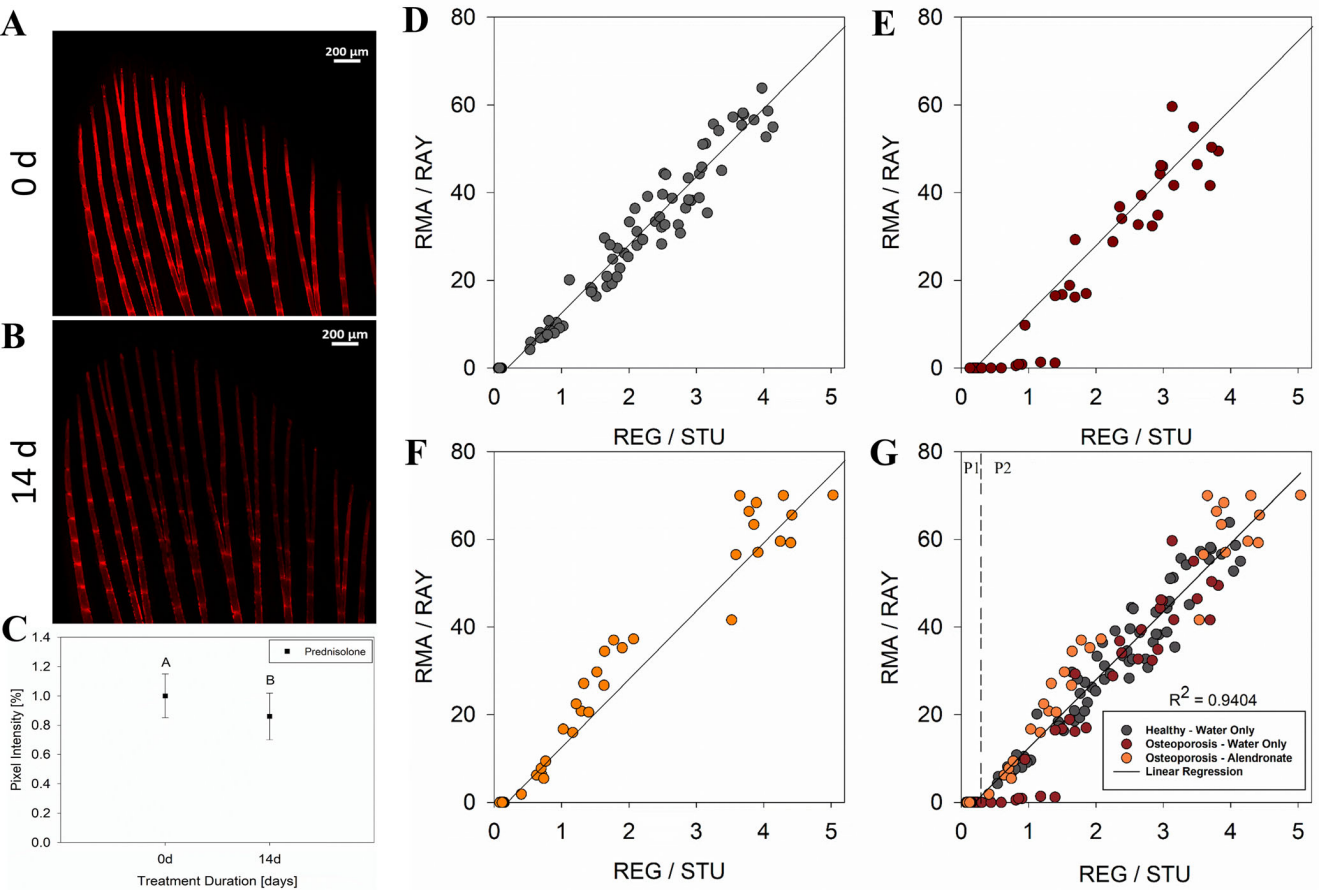
Glucocorticoid‐induced osteoporosis development and scatter plots showing the *de novo* mineralization vs. the overall regeneration. Lepidotrichia fluorescence signal before (*A*) and after (*B*) the treatment with PN. Representation of fluorescence intensities for all the fish analyzed (*C*). Healthy fish regenerating in fish tank water only (*D*); fish with osteoporosis phenotype regenerating in fish tank water only (*E*); fish with osteoporosis phenotype regenerating in fish tank water containing ALN (*F*); all treatments plotted in the same graphic with linear regression analysis showing the strong relationship between the variables (*G*). The P1 and P2 (linear increase) regions are displayed in “D” separated by the dashed line. R^2^ showed the strong and positive interaction between variables. Different capital letters indicate statistically significant difference (*p* < .01).

The pixel intensity (ie, mineralization level) of the images captured with confocal microscopy (Fig. 1*A,B*) were obtained and transformed quantitatively. The values prior to and after treatment with PN were plotted (Fig. 1*C*). Notably, the zebrafish treated with the glucocorticoid presented a significant drop on their caudal fin bony rays' fluorescence intensity (*p* < .01). The decreased signal intensity indicates lower mineralized tissue density, which is a characteristic of the bones with osteoporotic phenotype. The effect of medicine on the *de novo* mineralization of zebrafish caudal fins are shown in the various scatter plots of Fig. 1*D*–*G*. It is apparent that the behavior of the regenerative process is dictated by the different conditions used (Fig. 1*D*–*F*). Also, two different cluster regions were clearly noticed in all plots: in a first phase (P1), the values were all grouped at the *y* axis origin (RMA/RAY = 0), and in a second phase (P2), a linear increase were observed (RMA/RAY >0).

The time‐lapsed tracking of the caudal fin regeneration on nontreated subjects are displayed in the online supporting material (Supplementary Fig. S2). The bright field, fluorescence and merged columns represent the caudal fin regeneration, the *de novo* mineralization and the overall regenerative process, respectively. The first mineral deposits within the regenerating tissue were stained by AR‐S at 4 dpa. Fish subjects from the CTRL group displayed their first lepidotrichia bifurcation at 4 dpa.

### Ultrastructural and compositional analysis of zebrafish bone

**Fig 2:**
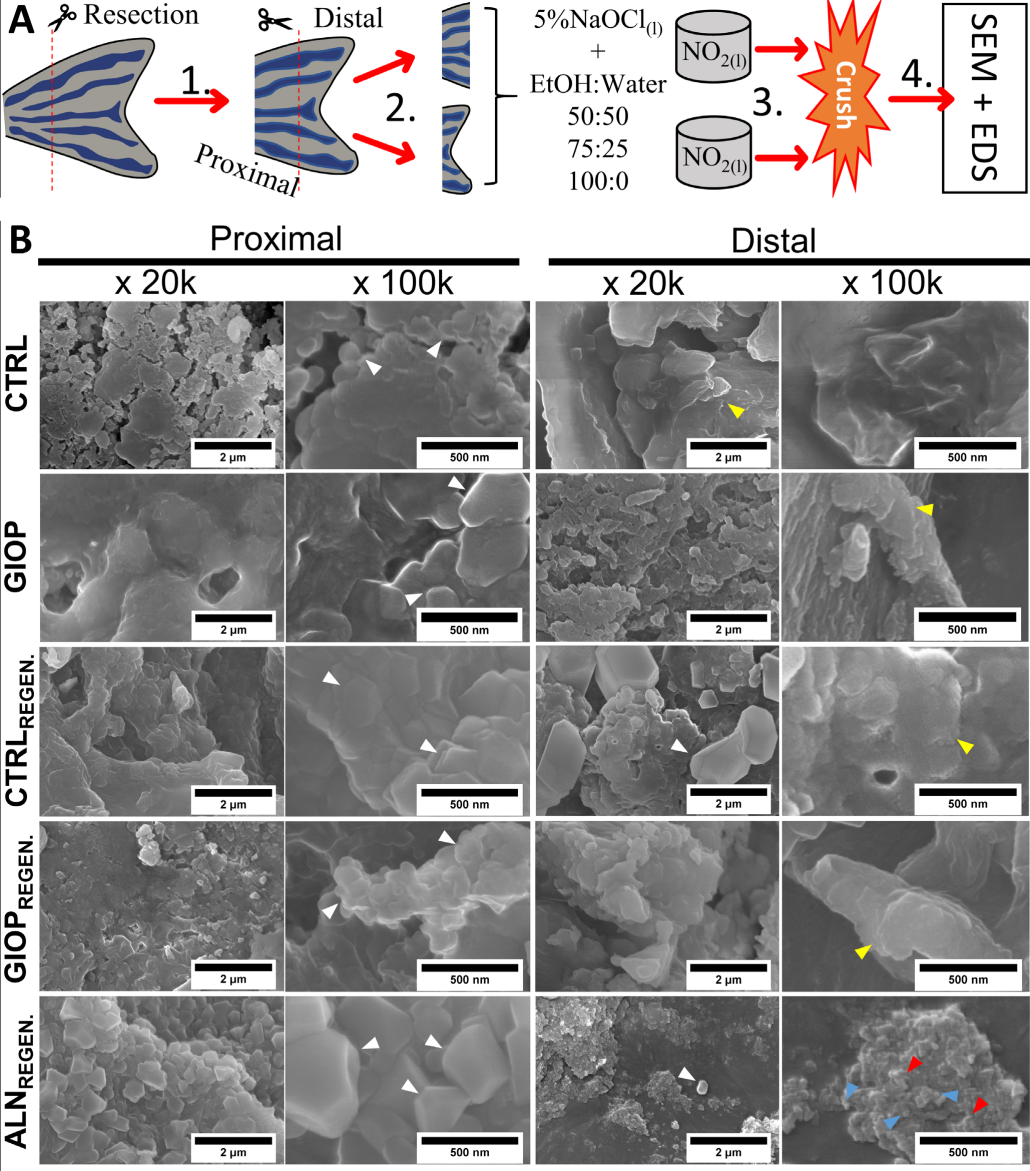
Scheme and SEM images of proximal and distal crushed bones extracted from zebrafish caudal fins before and after the proposed treatments, in ×20 k and ×100 k magnifications. (*A*) Processes performed prior to SEM analysis. Proximal crushed parts showed minerals with bigger size and well‐defined shapes, which was not commonly found in distal counterparts (white arrowheads), while the distal sites showed minerals resembling amorphous phases (yellow arrowheads). (*B*) The distal region of ALN_REGEN_ showed grouped small spherical‐like (light blue arrowheads) and plate‐like morphologies (red arrowheads).

A scheme of the procedures prior SEM imaging are displayed in Fig. 2*A*. The ultrastructural features of crushed bony fin rays are shown in the Fig. 2*B*. Arrangements with well‐defined edges and organized in layered structures of minerals were predominant on the proximal regions of CTRL, CTRL_REGEN_, and ALN_REGEN_. The average diameter of the structures calculated for CTRL, CTRL_REGEN_, and ALN_REGEN_ were 102.49 ± 14.64 nm, 118.63 ± 8.43 nm, and 125.60 ± 10.38 nm, respectively. On distal zones, for all groups with the exception of ALN_REGEN_, it was observed that there was a prevalence of structures with grooves on the surface. On the other hand, for ALN_REGEN_ distal region, structures were organized in coexistent small spherical‐like and plate‐like shapes. The elemental content of the SEM images was verified by EDS, and the results are shown in Table 2. The Ca content of samples in the proximal region varied from 5.77 ± 1.64 (GIOP) to 27.23 ± 3.59 (CTRL) and the P, for the same region, varied from 3.96 ± 0.50 (GIOP) to 15.68 ± 1.42 (CTRL). In all groups but GIOP, a higher percentage of Ca was found in the proximal part of the amputated fin. Similar phenomenon was observed for the Ca/P ratio; statistically significant differences was observed for CTRL, GIOP, CTRL_REGEN_, and GIOP_REGEN_ when comparing proximal to distal parts (*p* < .01). The difference in the distinct parts of ALN_REGEN_, however, was not significant (*p* > .01). The highest significant value was obtained for CTRL (1.74 ± 0.10) and CTRL_REGEN_ (1.56 ± 0.06) in the proximal region (*p* < .01).

**Table 2:** EDS Results (mean ± SD) for the Proximal and Distal Bones Extracted from Zebrafish Caudal Fins

	Elements [atomic%]					
	Proximal		Distal		Ca/P ratio	
Group	Ca	P	Ca	P	Proximal	Distal
CTRL	27.23 ± 3.59	15.68 ± 1.42	7.78 ± 1.22	5.35 ± 0.79	1.74 ± 0.10^Aa^	1.45 ± 0.05^Ab^
GIOP	5.77 ± 1.64	3.96 ± 0.50	6.25 ± 1.19	4.96 ± 0.92	1.46 ± 0.04^Ba^	1.26 ± 0.01^Bb^
CTRL_REGEN_	11.85 ± 0.76	7.60 ± 0.31	8.28 ± 0.40	6.34 ± 0.27	1.56 ± 0.06^ABa^	1.31 ± 0.03^ABb^
GIOP_REGEN_	10.11 ± 2.19	7.39 ± 1.51	5.28 ± 2.26	4.22 ± 1.31	1.37 ± 0.02^Ba^	1.25 ± 0.21^Bb^
ALN_REGEN_	11.57 ± 1.41	7.78 ± 0.83	4.46 ± 0.81	3.24 ± 0.67	1.49 ± 0.03^Ba^	1.38 ± 0.04^ABa^

Capital letters mean significant statistical difference in the same column (*p* < .01). Small letters mean significant difference in the same row (*p* < .01)

### Calcium‐phosphate profile

**Fig 3:**
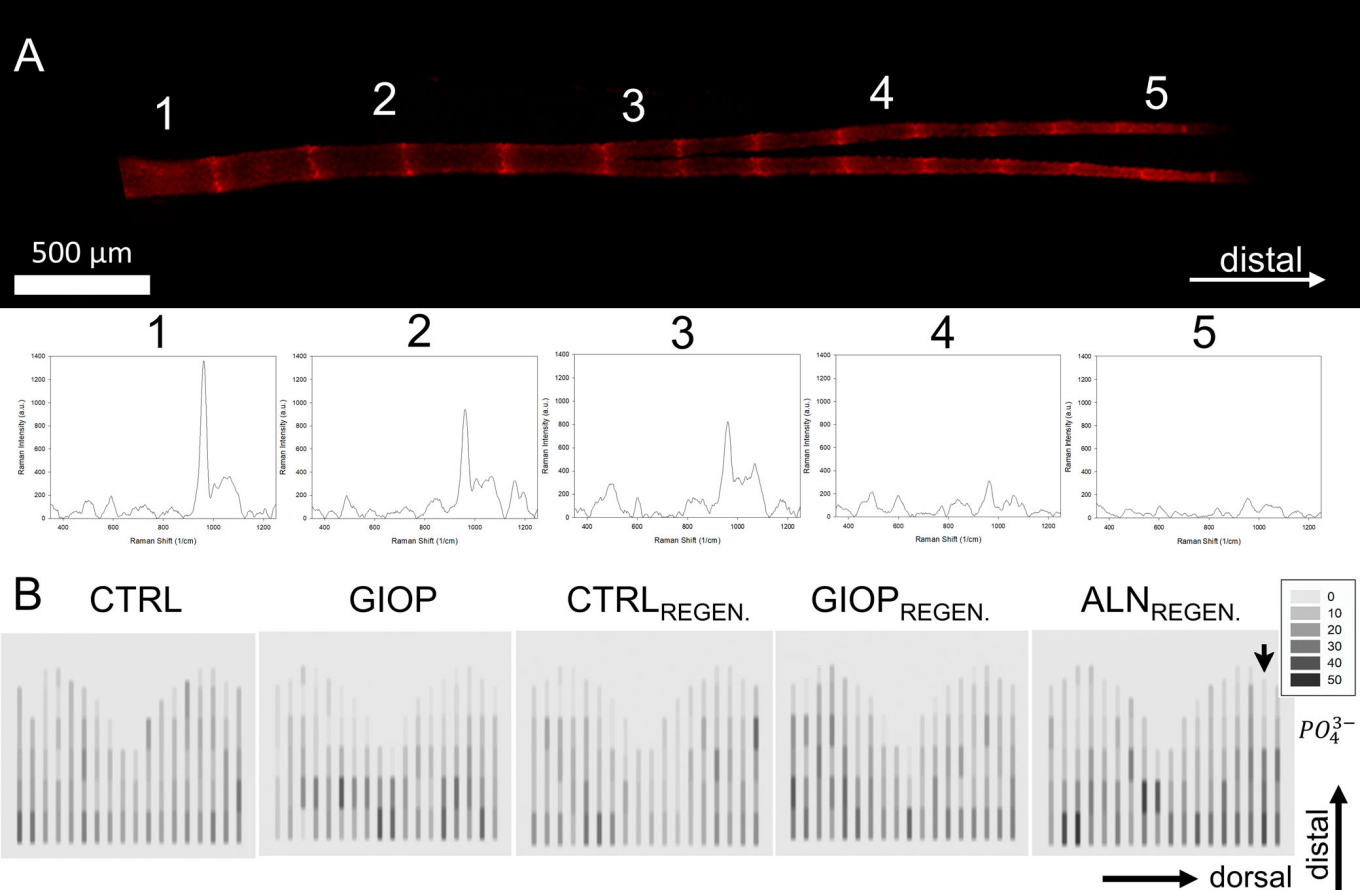
Montage containing a representative zebrafish caudal fin bony ray and the Raman spectra obtained. (*A*) Representation of the 17th lepidotrichia (dorsal region of the fin) of the ALN_REGEN_ group isolated from the fluorescence images obtained in confocal microscope; five different spots of interest along the growth direction were measured (*A*1–5) and the respective Raman peaks were obtained for each region. (*B*) Heat color plots representing the total area below the curve for the phosphate peak of ~962 cm^−1^; the black arrow indicates the bony ray represented in the Raman spectra shifts above. The legend box shows the total values of the areas divided by 10^3^. The darker the spots, the higher the mineral intensity of the signal.

Raman spectroscopy was used to analyze the intensity of the ν1 ${\mathrm{PO}}_{4}^{3-}$ peak (~960 cm^−1^) in each of the zebrafish caudal fin bony rays, from proximal region to the distal tips (Fig. 3*A*). Three phosphate and one carbonate peaks showed to be characteristic of zebrafish appendage bones: ν_1_ ${\mathrm{PO}}_{4}^{3-}$, ν_2_ ${\mathrm{PO}}_{4}^{3-}$, ν_4_ ${\mathrm{PO}}_{4}^{3-}$, and ${\mathrm{CO}}_{3}^{2-}$. The area below the ν_1_ ${\mathrm{PO}}_{4}^{3-}$ peak was calculated for each of the 96 sites measured, and heatmaps representing each of the 18 lepidotrichia were produced (Fig. 3*B*). From proximal (A1) to distal (A5), it is clear that the ν_1_ ${\mathrm{PO}}_{4}^{3-}$ intensity decreases as the distance from the resection plane increases. However, the decreased gradient of intensity was not observed in all bony rays. Moreover, CRTL_REGEN_ and GIOP_REGEN_ presented lower intensities at the distal parts in comparison to other groups, indicating that the newly formed bones display lower ν_1_ ${\mathrm{PO}}_{4}^{3-}$ intensities. ALN_REGEN_ presented the highest area below the curve from all samples in their proximal region.

### Surface topography and roughness

**Fig 4:**
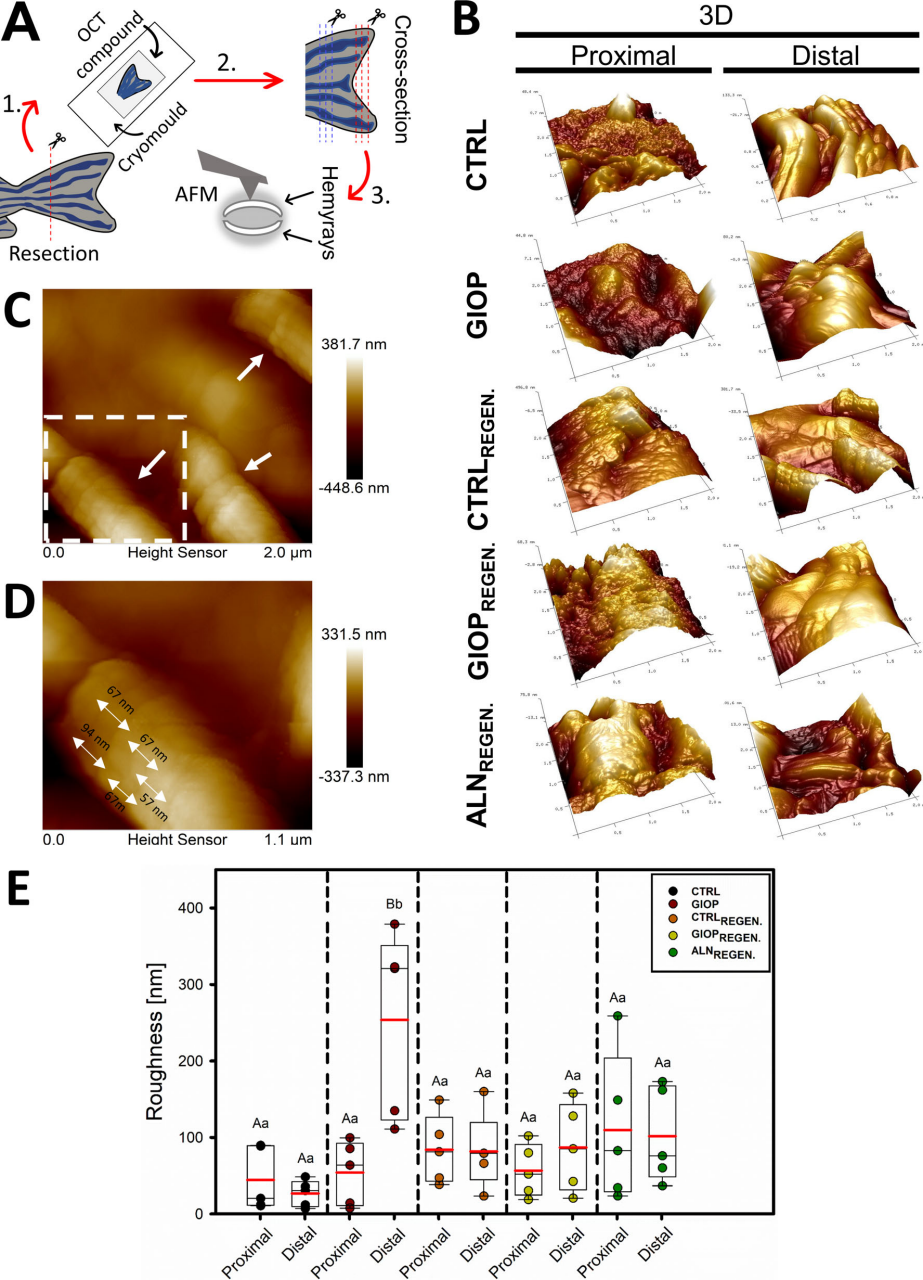
Surface topography profile of zebrafish bony rays and roughness calculation. (*A*) Procedure steps from the resection of the zebrafish caudal fins, to the analysis with AFM in tapping‐mode. (*B*) 3D representation of the cross‐section slices (obtained by cryosection process) analyzed by AFM. (*C*) The distal part of CTRL_REGEN_ group showing collagen fibers bundles (white single arrows) in flattened representations. (*D*) Approximation of the collagen fiber bundle from the white square in “B”; the double arrows shows the diameter of a periodic unit from a fibril (d ~ 75 nm). (*E*) Box‐plots with means (red horizontal line) and median (black horizontal line) of the roughness obtained from proximal and distal parts of fins by AFM. Capital letters indicate significant statistically difference between the different regions (proximal and distal) within the same treatment (*p* < .01). Lowercase letters indicate significant statistically difference between the same region (proximal or distal) among different treatments (*p* < .01).

Figure 4*A* shows the methodology used to obtain the sliced samples. The 3D surface profiles recorded for the proximal and distal caudal fin cross‐sections are represented in Fig. 4*B*. In general, the proximal regions of all groups have displayed densely‐packed structures on their surfaces, with small and abrupt bumps. On the other hand, at distal locations, the previously mentioned irregular profile, was substituted by comparatively less frequent irregularities. On distal sites collagen fibrils were found emerging on the surface of a less mineralized area (Fig. 4*C*). The collagen fibers were found to exhibit a periodicity range from 57 nm to 94 nm (Fig. 4*D*). From the acquired images, the mean value of surface roughness was then calculated based on the Ra parameter; the results are described in Fig. 4*E*. No statistically significant difference was observed among groups for proximal region (*p* > .01). However, the distal region of GIOP group presented significant higher roughness compared both with their proximal part and to all other distal regions evaluated (*p* < .01).

### Mechanical properties

**Fig 5:**
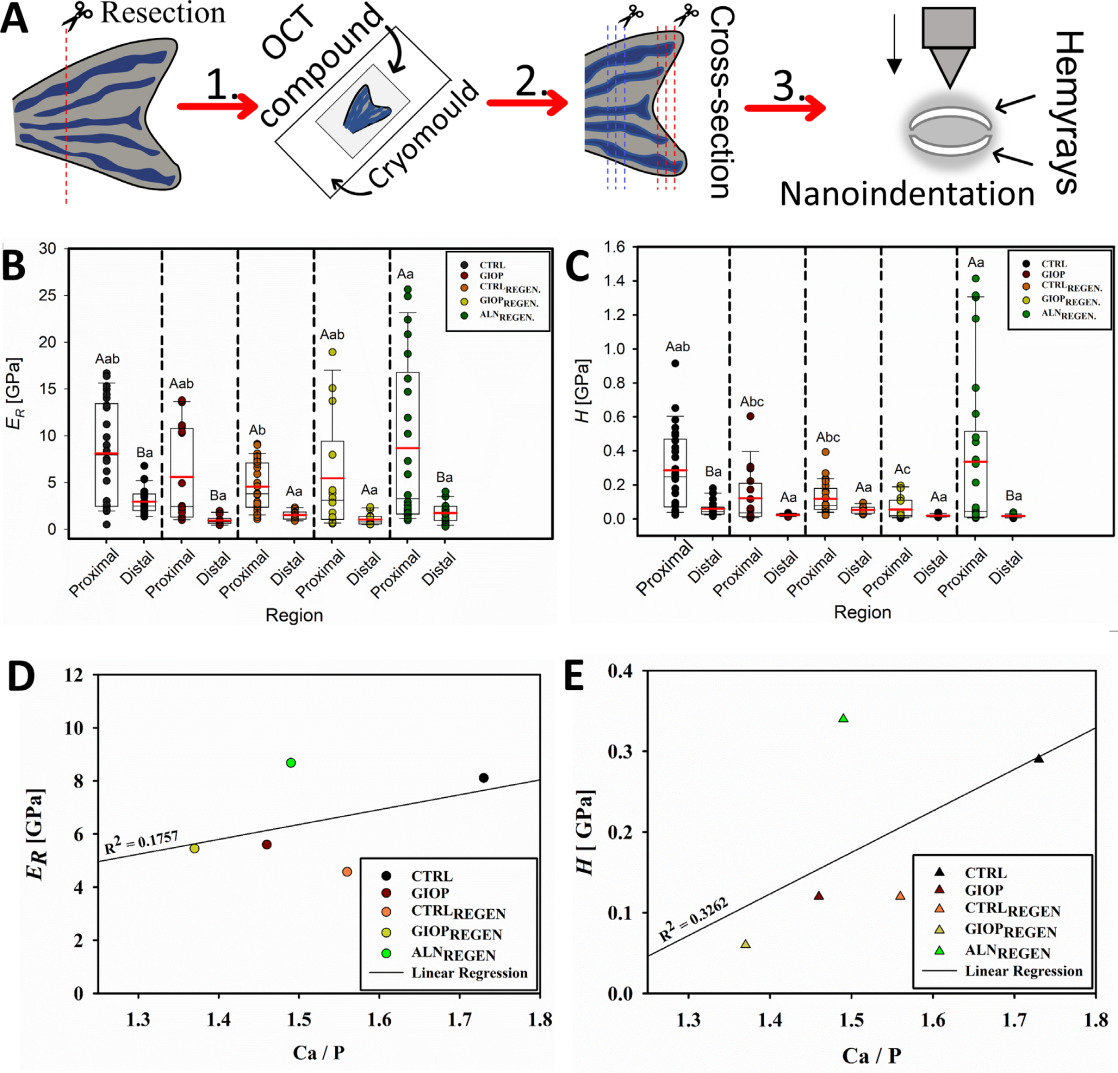
Mechanical performance of zebrafish caudal fin bony rays. (*A*) Procedure steps to obtain the caudal fin's slices for mechanical evaluation. Box‐plots with means (red horizontal line) and median (black horizontal line) of the *E*_*r*_ (*B*) and *H* (*C*) obtained from proximal and distal parts of fins with a triboindenter. Capital letters indicate significant statistically difference between the different regions (proximal and distal) within the same treatment (*p* < .01). Lowercase letters indicate significant statistically difference between the same region (proximal or distal) among different treatments (*p* < .01). The average values of the proximal parts of both *E*_*r*_ (*D*) and *H* (*E*) plotted against the Ca/P ratio. The regression lines show a weak but positive correlation between the variables.

The effects of PN and ALN on zebrafish caudal fin bony rays were also tested regarding the mechanical properties. Figure 5A summarizes the cross‐section procedure. The values of the *E*_*r*_ (Fig. 5*B*) and *H* (Fig. 5*C*), both expressed in GPa, obtained at proximal and distal bones of the fins. By means of *E*_*r*_ values, the proximal part of CTRL, GIOP, and ALN_REGEN_ were significantly higher than CTRL_REGEN_ and GIOP_REGEN_ (*p* < .01). If the values obtained at the proximal locations are compared within each other, the highest statistically significant value obtained was 8.68 ± 8.74 GPa (ALN_REGEN_) and the lowest was 4.57 ± 2.62 GPa (CTRL_REGEN_) (*p* < .01). By means of *H*, however, the proximal sites of both CTRL and ALN_REGEN_ (0.29 ± 0.23 GPa and 0.34 ± 0.47 GPa, respectively), were significantly higher than the distal parts (0.06 ± 0.05 GPa and 0.02 ± 0.01 GPa, respectively) (*p* < .01). The high standard deviations found (eg, ALN_REGEN_) may be related to the impossibility to perform finishing procedures on sectioned samples. Even though, statistically significant values were found. Also, both mechanical testing values were plotted in relation to the mineral ratio (Fig. 5*D,E*). The two (*E*_*r*_ and *H*), despite showing a weak dependence to Ca/P (*R* ^2^ equal to 0.1757 and 0.3262, respectively), showed a positive correlation.

## Discussion

In this work, we have implemented materials science principles to assess the properties of healthy, osteoporotic, and ALN‐treated zebrafish bones at the multiscale level. The fish were anesthetized with a cocktail of Tricaine and isoflurane in order to minimize the side effects of the dopant.^(^ ^32^, ^37^ ^)^ The fish had their fins resected and were left to regenerate during 21 days in water tanks with or without ALN. A second resection was performed and the effects of the various treatments were tested with a series of structural, elemental, and mechanical analysis. In agreement to previous research findings on higher vertebrate animal models, our study verified the reproducibility and reliability of the zebrafish model on the study of mineralized tissues. PN and ALN significantly affected the fin bones properties in comparison to the controls. Our data showed that ALN was able to restore the overall properties of zebrafish caudal fin osteoporotic bones to the levels of untreated fish. Interestingly, the bone's appearance seems to be more affected by the region that the minerals were extracted (proximal or distal) than by the treatment proposed, while the Ca/P ratio showed difference in both situations. This reinforces the benefits of materials science approach to evaluate diseased mineralized structures in a multiscale level. These findings provide insights in new strategies to evaluate diseased mineralized tissues, especially on those with osteoporotic phenotypes and drug action analysis. The null hypothesis was rejected.

The lepidotrichia of zebrafish is considered a powerful model to evaluate the various aspects of bone remodeling. Their ability to remineralize after resection is widely known.^(^ ^20^, ^24^ ^)^ We have thus used resection/regeneration protocols to exacerbate the drug effect before collecting the samples for analysis. By using the glucocorticoid‐induced osteoporosis protocol proposed in this work, the fluorescence intensity of caudal fin bones displayed a ~15% reduction within 2 weeks, which configures the development of osteoporotic‐like phenotype. In rodents, however, the development of osteoporosis may need increased time, depending on the bone analyzed.^(^ ^38^ ^)^ Hence, our evaluation protocol was rapid and sensitive enough to obtain significant results. Our data reconfirm the reliability of adult zebrafish caudal fin to be used as a reliable model to verify glucocorticoid‐induced osteoporosis.^(^ ^2^ ^)^

In 2016, Cardeira and colleagues^(^ ^20^ ^)^ suggested analyzing the caudal fin *de novo* mineralization by calculating the parameters (RMA, RAY, REG, and STU) of many zebrafish individuals during caudal fin regeneration. Here, the drugs' aftereffects in the lepidotrichia could be clearly observed with the methodology proposed. The scatter behavior plotted for each treatment was strongly related to the type of medicine given to the fish. The group with no intervention during the regenerative process, had the values calculated distributed above and below the linear regression line. This may represent the natural variation of each fish when no external elements interfere in the regenerative process. Any deviation from the regression line should indicate effects of stimulation (points above) or halt (points below) of the mineral deposition.^(^ ^20^ ^)^ Corroborating with studies that demonstrate PN as an anti‐mineralogenic compound,^(^ ^15^, ^16^, ^17^, ^39^ ^)^ we found lower values for the group that received no further intervention after developing osteoporotic‐like phenotype (values below regression line). In contrast, most of values for fish that received the pro‐mineralogenic compound (ALN), were shown above the same line (overmineralization). Our after‐effect plots suggest that this methodology may be useful for the testing different anti‐mineralogenic and pro‐mineralogenic compounds, and reconfirm PN and ALN to decrease and increase, respectively, the mineral deposition within zebrafish bones. The in vivo approach of time‐lapsed analysis on the same subject, may have also contributed to enhance the evidence strength of our results. Considering the potential of zebrafish models, we have reconfirmed zebrafish as a suitable organism for the evaluation of bone mineralization, which could enable reduced use of rodents in future research.^(^ ^22^ ^)^

The *de novo* mineralization of zebrafish caudal fins involve a series of complex events.^(^ ^24^, ^25^, ^26^ ^)^ From the initial bone formation to the development of the actinotrichia, the mineralized tissue is known to be composed of different structures, depending on the availability of Ca and P resources.^(^ ^33^ ^)^ The intake of mineralogenic compounds could then affect the zebrafish bones at a multiscale level.^(^ ^10^ ^)^ We have analyzed the appearance and mineral content of two distinct regions of the bony rays affected by PN or ALN, before and after the regrowth. The mineralized structures found in the proximal region are clearly different from the distal minerals; they presented well‐defined edges and contours. Similar morphologies were almost inexistent in the bones obtained from distal sites (Fig. 2*B*). The underlying mineralization process in the zebrafish caudal fins occurs from the proximal to the distal region by the addition of newly‐formed minerals at the furthermost edge of each fin.^(^ ^33^ ^)^ The administration of medicines did not seem to have visibly affected the appearance of the minerals observed at the proximal regions. Interestingly, the fish that received ALN displayed coexistent small rounded and plate‐like shapes in the distal sites; similar structures could not be observed in high magnification SEM images in other treatment conditions. Coexisting round and plate‐like minerals were found in amorphous forms of calcium‐phosphates,^(^ ^33^, ^40^ ^)^ which are related to the first stages of mineralization.^(^ ^41^ ^)^ The mineralization course of events starts with the saturation of an amorphous calcium phosphate (ACP) phase, followed by the mineral deposition within the collagen fibers and subsequent crystal growth.^(^ ^40^, ^42^ ^)^ Moreover, the bone's natural layered structures were preserved.^(^ ^34^ ^)^ Similar structures have been reported in previous studies.^(^ ^33^ ^)^ Hence, our results suggest that the treatment with bisphosphonate do not reconfigure the natural morphology of bones during the 21 days of zebrafish appendage regeneration, but may have accelerated the transition between mineral development stages.

The mineral balance and bone remodeling potentials are intimately related to the Ca and P content in the site.^(^ ^34^ ^)^ In the conditions proposed in this work, a broad range of Ca, P, and mineral ratios were found in the fin bones. This finding corroborates with the concepts that different types of minerals coexist in zebrafish caudal fin bones.^(^ ^33^, ^40^ ^)^ The Ca/P proportion was found to significantly decrease in proximal‐distal direction. This behavior was observed for all but ALN_REGEN_ group. Higher mineral ratios are related with less pronounced effects of mineralogenic compounds in the proximal regions. On the other hand, lower mineral availability, represented by decreased Ca/P ratios, could be more influenced by both PN and ALN.^(^ ^43^ ^)^ This is shown by the significant lower mineral ratios found in the bones of fish after the intake of PN and the narrow difference between the proximal and distal parts of bones affected by ALN. Our multiscale analysis suggests that ALN increase the mineral ratio of ACP in vulnerable areas first. In the same manner, PN may affect the distal areas first, which enhances the vulnerability and may favor the binding of ALN to the bone.^(^ ^23^ ^)^

To completely understand the effects of medicines and diseases in mineralized tissues, the evaluation of characteristics in molecular level is essential.^(^ ^9^, ^44^ ^)^ The intensity of the peaks observed in Raman analysis was strongly related to the vibration of the molecules excited while the test was performed.^(^ ^14^, ^44^, ^45^ ^)^ Overall, the proximal regions displayed increased areas below the ~960 cm^−1^ peak. Our compositional analysis of Ca/P ratio has shown a great range of mineral ratios depending on the treatment and the mineral profile suggests a similar outcome. However, any difficulties related to the use of bulk characterization methods (eg, Raman spectroscopy) in mineralized tissues with strongly mixed crystalline and amorphous phases should not be ignored, and may also explain the results obtained.^(^ ^33^ ^)^

Osteoporotic‐like bones are characterized by the mineral resorption caused by increased osteoclasts activity that often reconfigure bone's microarchitecture.^(^ ^3^, ^13^, ^46^ ^)^ Following the overall trend in this research, the proximal areas verified by AFM revealed a different appearance from those obtained from distal sites. The frequent and low amplitude peaks images found in the bones extracted from the proximal regions are characteristic from highly mineralized tissues.^(^ ^42^, ^47^ ^)^ As we analyzed spots distant to the resection plane, a less mineralized characteristic was observed and collagen fibers were exposed (CTRL_REGEN_). The collagen fibers were presented with variable periodicity. The type I collagen commonly found in zebrafish bones is well known to have periodicity of 67 nm.^(^ ^48^ ^)^ Lower periodicity lengths are related to the early stages of minerals in zebrafish and, in favorable conditions, the periodicity increases its length.^(^ ^42^ ^)^ This reconfirm that the zebrafish bones are composed by different phases and development stages of Ca and P.^(^ ^33^, ^40^ ^)^

The osteoporotic phenotype is related to poor mechanical properties due to its mineral deficiency and higher porosity in comparison to sound bones.^(^ ^8^, ^12^ ^)^ The mechanical attributes of zebrafish bones should be intimately related to the microstructure and to the composition of the mineralized tissue.^(^ ^34^, ^42^, ^48^ ^)^ The highly mineralized structures found in the proximal bones showed, in general, increased *E*_*r*_ and *H*. At the distal regions, where the ratio of nonmineralized tissues rises, the surfaces were found to be softer, but not significantly lower for all the conditions proposed. In proximal bones, the reduction in *H* caused by the administration of PN was countered by the intake of ALN. The efficacy of bisphosphonates is strongly related to their affinity to bind to the bones and ability to suppress the farnesyl pyrophosphate synthase, essential to the natural pathways of osteoclasts.^(^ ^10^ ^)^ The regulation of the balance between ossification and bone resorption led to increased mineral levels, as we demonstrated with EDS. The pro‐mineralogenic compound was not only able to recover the *E*_*r*_ and *H* to the original values of healthy bones, but it also showed adequate increase, not surpassing significantly the natural value of nontreated individuals.^(^ ^9^ ^)^ Moreover, our results match with the current literature on zebrafish.^(^ ^34^, ^42^, ^48^ ^)^ This is especially important because zebrafish bones are small and novel approaches are needed to assess their properties. In order to verify the relationship between the mechanical analysis and bone's composition, we have calculated the value of the Pearson correlation. The *E*_*r*_ and *H* have showed a low, but positive, interaction with the Ca/P ratios of the proximal regions affected by the medicine. This outcome reaffirms the feasibility of a multiscale approach to study sound and diseased bones, affected or not, by mineralogenic compounds.

Our study fills a gap on the topic of diseased mineralized tissues using a time‐lapsed approach of following the same individual throughout the experimentation. To the best of our knowledge, this is the first study to use a series of materials science approaches, and correlate with zebrafish bones under the effect of PN and reconfirm ALN was able to rescue the osteoporotic phenotype of the fish, which was achieved by the restoration of the *E*_*r*_ and *H* to the normal range. However, although zebrafish is considered a powerful model to study bone‐related disease and mineralization, there are some limitations in their use that were already summarized^(^ ^23^, ^34^ ^)^: (i) the bone remodeling process differs from the mammals especially due to the presence of mononucleated and multinucleated osteoclasts^(^ ^23^ ^)^; (ii) the nature of the lepidotrichia is dermal ossification,^(^ ^27^ ^)^ which also mechanistically differs them from the mammals; (iii) despite a complete mapping of properties alongside the caudal fin bony rays were performed and comparisons between different single pieces of bones could be done, the variability in different individuals was not tested (eg, Raman mapping); (iv) moreover, zebrafish bones are under the effect of a different loading environment than non‐aquatic mammals.^(^ ^34^ ^)^ Thus, the results here proposed should be taken carefully. Despite that, the mechanical evaluation, in the conditions proposed, showed to be precise enough to assess zebrafish bones with different mineral levels. Our research could help to develop new strategies to evaluate overall quality of diseased mineralized tissues, especially on those with osteoporotic phenotypes and drug action analysis.

## Conclusions

Zebrafish caudal fin bony rays were shown to be a feasible and fast model to study the elemental, structural and mechanical characteristics of bones affected by anti‐mineralogenic and pro‐mineralogenic medicines. The administration of PN during the time proposed reduced in about 15% the fluorescence intensity of the zebrafish caudal fin bones. The anti‐mineralogenic medicine also showed to reduce the Ca/P of the distal bones to 1.26, whereas ALN was able to partially recover it to the untreated levels (Ca/P = 1.38) and increase the mineral organization. The mechanical behavior of the bones was also restored to the initial range by the pro‐mineralogenic compound. The presented approach may be used to test different compounds and have the potential to be used in newly developed drugs related to bone remodeling. Further research should be done regarding the long‐term intake of medicines or their dose dependence on bone properties.

### PEER REVIEW

The peer review history for this article is available at https://publons.com/publon/10.1002/jbm4.10435.

## Supporting information


**Supplementary Fig. S1** Flowchart with the experimental protocols used in this study.

**Supplementary Fig. S2** Bone and tissue regeneration process of zebrafish caudal fin. The bright field column represents the tissue growth; the fluorescence column represents the de novo mineralization process; the merged column images represent the overall process (tissue + mineral) regeneration. The scale bar sizes are 500 μm.

## References

[1] Rachner TD , Khosla S , Hofbauer LC . Osteoporosis: now and the future. Lancet. 2011;377(9773):1276–87. 21450337 10.1016/S0140-6736(10)62349-5 PMC3555696

[2] Lin Y , Xiang X , Chen T , et al. In vivo monitoring and high‐resolution characterizing of the prednisolone‐induced osteoporotic process on adult zebrafish by optical coherence tomography. Biomed Opt Express. 2019;10(3):1184. 30891338 10.1364/BOE.10.001184 PMC6420289

[3] Chavassieux P , Chapurlat R , Portero‐Muzy N , et al. Bone‐forming and antiresorptive effects of romosozumab in postmenopausal women with osteoporosis: bone histomorphometry and microcomputed tomography analysis after 2 and 12 months of treatment. J Bone Miner Res. 2019;34(9):1597–608. 31233639 10.1002/jbmr.3735 PMC7027577

[4] Dreinhöfer KE , Mitchell PJ , Bégué T , et al. A global call to action to improve the care of people with fragility fractures. Injury. 2018;49(8):1393–7. 29983172 10.1016/j.injury.2018.06.032

[5] Odén A , McCloskey EV , Kanis JA , Harvey NC , Johansson H . Burden of high fracture probability worldwide: secular increases 2010–2040. Osteoporos Int. 2015;26(9):2243–8. 26018089 10.1007/s00198-015-3154-6

[6] Cheng T‐T , Lai H‐M , Yu S‐F , et al. The impact of low‐dose glucocorticoids on disease activity, bone mineral density, fragility fractures, and 10‐year probability of fractures in patients with rheumatoid arthritis. J Invest Med. 2018;66(6):1004–7. 10.1136/jim-2018-000723 PMC6073913 29891493

[7] Ma X , Xu Z , Ding S , Yi G , Wang Q . Alendronate promotes osteoblast differentiation and bone formation in ovariectomy‐induced osteoporosis through interferon‐β/signal transducer and activator of transcription 1 pathway. Exp Ther Med. 2017;15(1):182–90. 29375681 10.3892/etm.2017.5381 PMC5763659

[8] Bernhard A , Milovanovic P , Zimmermann EA , et al. Micro‐morphological properties of osteons reveal changes in cortical bone stability during aging, osteoporosis, and bisphosphonate treatment in women. Osteoporos Int. 2013;24(10):2671–80. 23632826 10.1007/s00198-013-2374-x

[9] Hassler N , Gamsjaeger S , Hofstetter B , Brozek W , Klaushofer K , Paschalis EP . Effects of long‐term alendronate treatment on postmenopausal osteoporosis bone material properties. Osteoporos Int. 2015;26(1):339–52. 25315260 10.1007/s00198-014-2929-5

[10] Russell RGG . Bisphosphonates: mode of action and pharmacology. Pediatrics. 2007;119(Suppl 2):S150–62. 17332236 10.1542/peds.2006-2023H

[11] Iba K , Takada J , Sonoda T , Yamashita T . Effect of continuous long‐term treatment for 10 years with bisphosphonate on Japanese osteoporosis patients. J Bone Miner Metab. 2020;38(2):240–7. 31667583 10.1007/s00774-019-01049-1

[12] Loundagin LL , Haider IT , Cooper DML , Edwards WB . Association between intracortical microarchitecture and the compressive fatigue life of human bone: a pilot study. Bone Rep. 2020;12:100254. 32258250 10.1016/j.bonr.2020.100254 PMC7110329

[13] Kim G‐J , Yoo HS , Lee KJ , Choi JW , Hee An J . Image of the micro‐computed tomography and atomic‐force microscopy of bone in osteoporosis animal model. J Nanosci Nanotechnol. 2018;18(10):6726–31. 29954487 10.1166/jnn.2018.15472

[14] Bennet M , Akiva A , Faivre D , et al. Simultaneous Raman microspectroscopy and fluorescence imaging of bone mineralization in living zebrafish larvae. Biophys J. 2014;106(4):L17–9. 24560001 10.1016/j.bpj.2014.01.002 PMC3944822

[15] Barrett R , Chappell C , Quick M , Fleming A . A rapid, high content,in vivo model of glucocorticoid‐induced osteoporosis. Biotechnol J. 2006;1(6):651–5. 16892313 10.1002/biot.200600043

[16] Bergen DJM , Kague E , Hammond CL . Zebrafish as an emerging model for osteoporosis: a primary testing platform for screening new osteo‐active compounds. Front Endocrinol. 2019;10:6. 10.3389/fendo.2019.00006 PMC6361756 30761080

[17] de Vrieze E , van Kessel MAHJ , Peters HM , Spanings FAT , Flik G , Metz JR . Prednisolone induces osteoporosis‐like phenotype in regenerating zebrafish scales. Osteoporos Int. 2014;25(2):567–78. 23903952 10.1007/s00198-013-2441-3

[18] Schönbörner AA , Boivin G , Baud CA . The mineralization processes in teleost fish scales. Cell Tissue Res. 1979;202(2):203–12. 519703 10.1007/BF00232235

[19] Pasqualetti S , Congiu T , Banfi G , Mariotti M . Alendronate rescued osteoporotic phenotype in a model of glucocorticoid‐induced osteoporosis in adult zebrafish scale. Int J Exp Pathol. 2015;96(1):11–20. 25603732 10.1111/iep.12106 PMC4352348

[20] Cardeira J , Gavaia PJ , Fernández I , et al. Quantitative assessment of the regenerative and mineralogenic performances of the zebrafish caudal fin. Sci Rep. 2016;6(1):39191. 27991522 10.1038/srep39191 PMC5171864

[21] Bensimon‐Brito A , Cardeira J , Dionísio G , Huysseune A , Cancela ML , Witten PE . Revisiting in vivo staining with alizarin red S—a valuable approach to analyse zebrafish skeletal mineralization during development and regeneration. BMC Dev Biol. 2016;16(1):2. 26787303 10.1186/s12861-016-0102-4 PMC4719692

[22] Bruneel B , Witten PE . Power and challenges of using zebrafish as a model for skeletal tissue imaging. Connect Tissue Res. 2015;56(2):161–73. 25689092 10.3109/03008207.2015.1013193

[23] Geurtzen K , Vernet A , Freidin A , et al. Immune suppressive and bone inhibitory effects of prednisolone in growing and regenerating zebrafish tissues. J Bone Miner Res. 2017;32(12):2476–88. 28771888 10.1002/jbmr.3231

[24] Pfefferli C , Jaźwińska A . The art of fin regeneration in zebrafish: the art of fin regeneration. Regeneration. 2015;2(2):72–83. 27499869 10.1002/reg2.33 PMC4895310

[25] Uemoto T , Abe G , Tamura K . Regrowth of zebrafish caudal fin regeneration is determined by the amputated length. Sci Rep. 2020;10(1):649. 31959817 10.1038/s41598-020-57533-6 PMC6971026

[26] Azevedo AS , Grotek B , Jacinto A , Weidinger G , Saúde L . The regenerative capacity of the zebrafish caudal fin is not affected by repeated amputations. PLoS One. 2011;6(7):e22820. 21829525 10.1371/journal.pone.0022820 PMC3145768

[27] Geurtzen K , Knopf F , Wehner D , Huitema LFA , Schulte‐Merker S , Weidinger G . Mature osteoblasts dedifferentiate in response to traumatic bone injury in the zebrafish fin and skull. Development. 2014;141(11):2225–34. 24821985 10.1242/dev.105817

[28] Knopf F , Hammond C , Chekuru A , et al. Bone regenerates via dedifferentiation of osteoblasts in the zebrafish fin. Dev Cell. 2011;20(5):713–24. 21571227 10.1016/j.devcel.2011.04.014

[29] Geurtzen K , Knopf F . Adult zebrafish injury models to study the effects of prednisolone in regenerating bone tissue. J Vis Exp. 2018;140:e58429. 10.3791/58429 PMC6235570 30394396

[30] Embry MR , Belanger SE , Braunbeck TA , et al. The fish embryo toxicity test as an animal alternative method in hazard and risk assessment and scientific research. Aquat Toxicol. 2010;97(2):79–87. 20061034 10.1016/j.aquatox.2009.12.008

[31] Westerfield M . The zebrafish book. A guide for the laboratory use of zebrafish (Danio rerio). 3rd ed Eugene, OR: University of Oregon Press; 1995.

[32] Huang W‐C , Hsieh Y‐S , Chen I‐H , et al. Combined use of MS‐222 (Tricaine) and isoflurane extends anesthesia time and minimizes cardiac rhythm side effects in adult zebrafish. Zebrafish. 2010;7(3):297–304. 20807039 10.1089/zeb.2010.0653

[33] Mahamid J , Sharir A , Addadi L , Weiner S . Amorphous calcium phosphate is a major component of the forming fin bones of zebrafish: indications for an amorphous precursor phase. Proc Natl Acad Sci U S A. 2008;105(35):12748–53. 18753619 10.1073/pnas.0803354105 PMC2529085

[34] Chang Z , Chen P‐Y , Chuang Y‐J , Akhtar R . Zebrafish as a model to study bone maturation: nanoscale structural and mechanical characterization of age‐related changes in the zebrafish vertebral column. J Mech Behav Biomed Mater. 2018;84:54–63. 29747057 10.1016/j.jmbbm.2018.05.004

[35] Fischer‐Cripps AC . A review of analysis methods for sub‐micron indentation testing. Vacuum. 2000;58(4):569–85.

[36] Chazotte B . Labeling Golgi with fluorescent ceramides. Cold Spring Harb Protoc. 2012;2012(8):pdb.prot070599. 22854569 10.1101/pdb.prot070599

[37] Collymore C , Tolwani A , Lieggi C , Rasmussen S . Efficacy and safety of 5 anesthetics in adult zebrafish (*Danio rerio*). J Am Assoc Lab Anim Sci. 2014;53(2):198–203. 24602548 PMC3966278

[38] Yousefzadeh N , Kashfi K , Jeddi S , Ghasemi A . Ovariectomized rat model of osteoporosis: a practical guide. EXCLI J. 2020;19:89–107. 32038119 10.17179/excli2019-1990 PMC7003643

[39] He H , Wang C , Tang Q , Yang F , Xu Y . Possible mechanisms of prednisolone‐induced osteoporosis in zebrafish larva. Biomed Pharmacother. 2018;101:981–7. 29635908 10.1016/j.biopha.2018.02.082

[40] Mahamid J , Aichmayer B , Shimoni E , et al. Mapping amorphous calcium phosphate transformation into crystalline mineral from the cell to the bone in zebrafish fin rays. Proc Natl Acad Sci U S A. 2010;107(14):6316–21. 20308589 10.1073/pnas.0914218107 PMC2851957

[41] Mahamid J , Addadi L , Weiner S . Crystallization pathways in bone. Cells Tissues Organs. 2011;194(2–4):92–7. 21576906 10.1159/000324229

[42] Ge J , Wang X , Cui F . Microstructural characteristics and nanomechanical properties across the thickness of the wild‐type zebrafish skeletal bone. Mater Sci Eng C. 2006;26(4):710–5.

[43] de Vrieze E , Heijnen L , Metz JR , Flik G . Evidence for a hydroxyapatite precursor in regenerating cyprinid scales: hydroxyapatite precursor in regenerating scales. J Appl Ichthyol. 2012;28(3):388–92.

[44] Toledano M , Toledano‐Osorio M , Guerado E , Caso E , Aguilera FS , Osorio R . Biochemical assessment of nanostructures in human trabecular bone: proposal of a Raman microspectroscopy based measurements protocol. Injury. 2018;49:S11–21. 30077357 10.1016/j.injury.2018.07.034

[45] Morris MD , Mandair GS . Raman assessment of bone quality. Clin Orthop Relat Res. 2011;469(8):2160–9. 21116756 10.1007/s11999-010-1692-y PMC3126952

[46] Rolvien T , Milovanovic P , Schmidt FN , et al. Long‐term immobilization in elderly females causes a specific pattern of cortical bone and osteocyte deterioration different from postmenopausal osteoporosis. J Bone Miner Res. 2020;35(7):1343–51. 31999373 10.1002/jbmr.3970

[47] Dorozhkin SV , Epple M . Biological and medical significance of calcium phosphates. Angew Chem Int Ed. 2002;41(17):3130–46. 10.1002/1521-3773(20020902)41:17<3130::AID-ANIE3130>3.0.CO;2-1 12207375

[48] Wang L , Liu B , Li H , et al. Long‐range ordered carbon clusters: A crystalline material with amorphous building blocks. Science. 2012;337(6096):825–8. 22904007 10.1126/science.1220522

