# Supplementary figures and images for: Influence of Prednisolone and Alendronate on the de novo Mineralization of Zebrafish Caudal Fin
Source: JBMR Plus. 2020 Dec 5;5(2):e10435. doi: 10.1002/jbm4.10435 (PMC7872341; doi:10.1002/jbm4.10435)

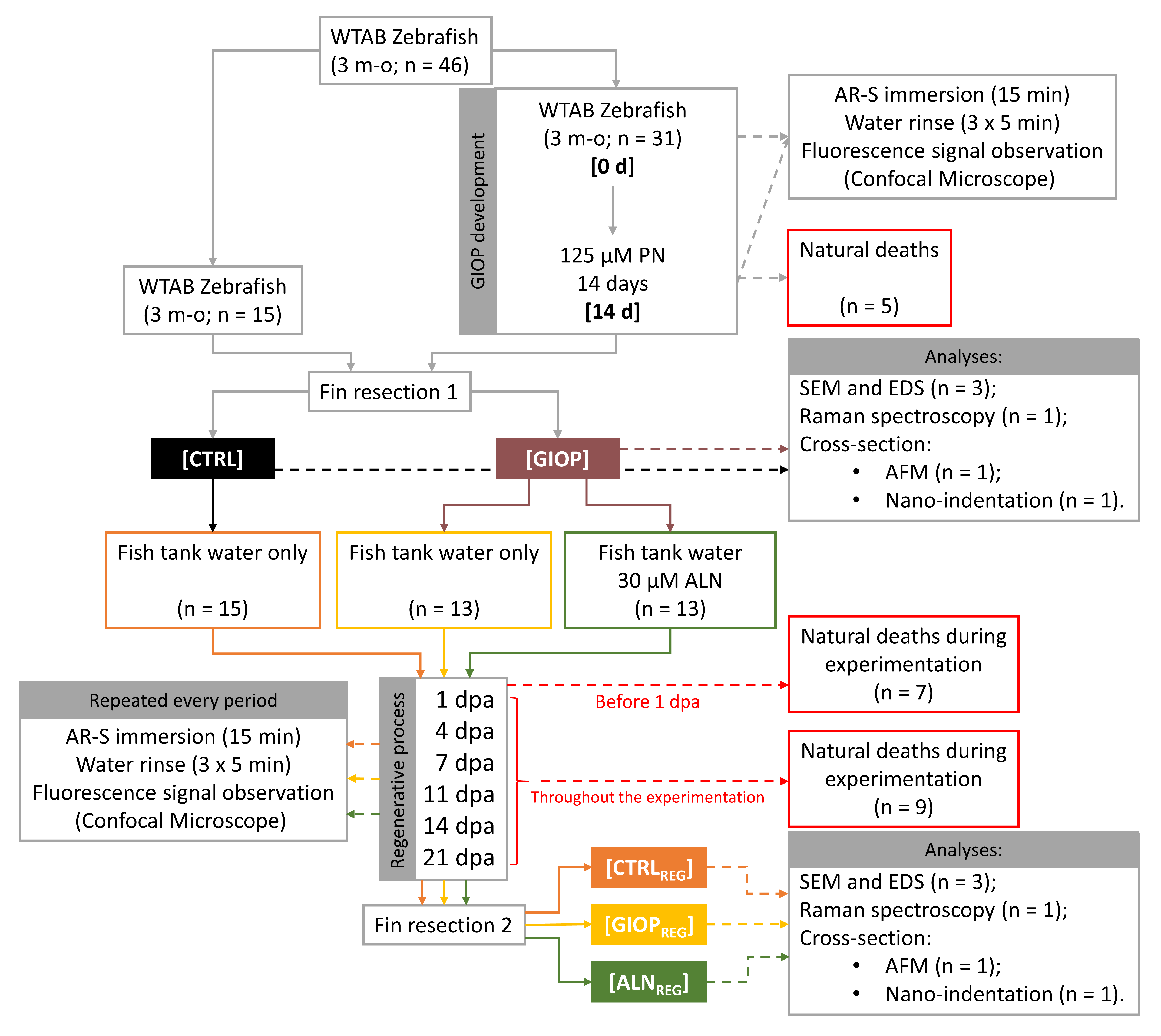

Supplement: Supplementary file 1 — Supplementary Fig. S1 Flowchart with the experimental protocols used in this study. [file JBM4-5-e10435-s001.tif]

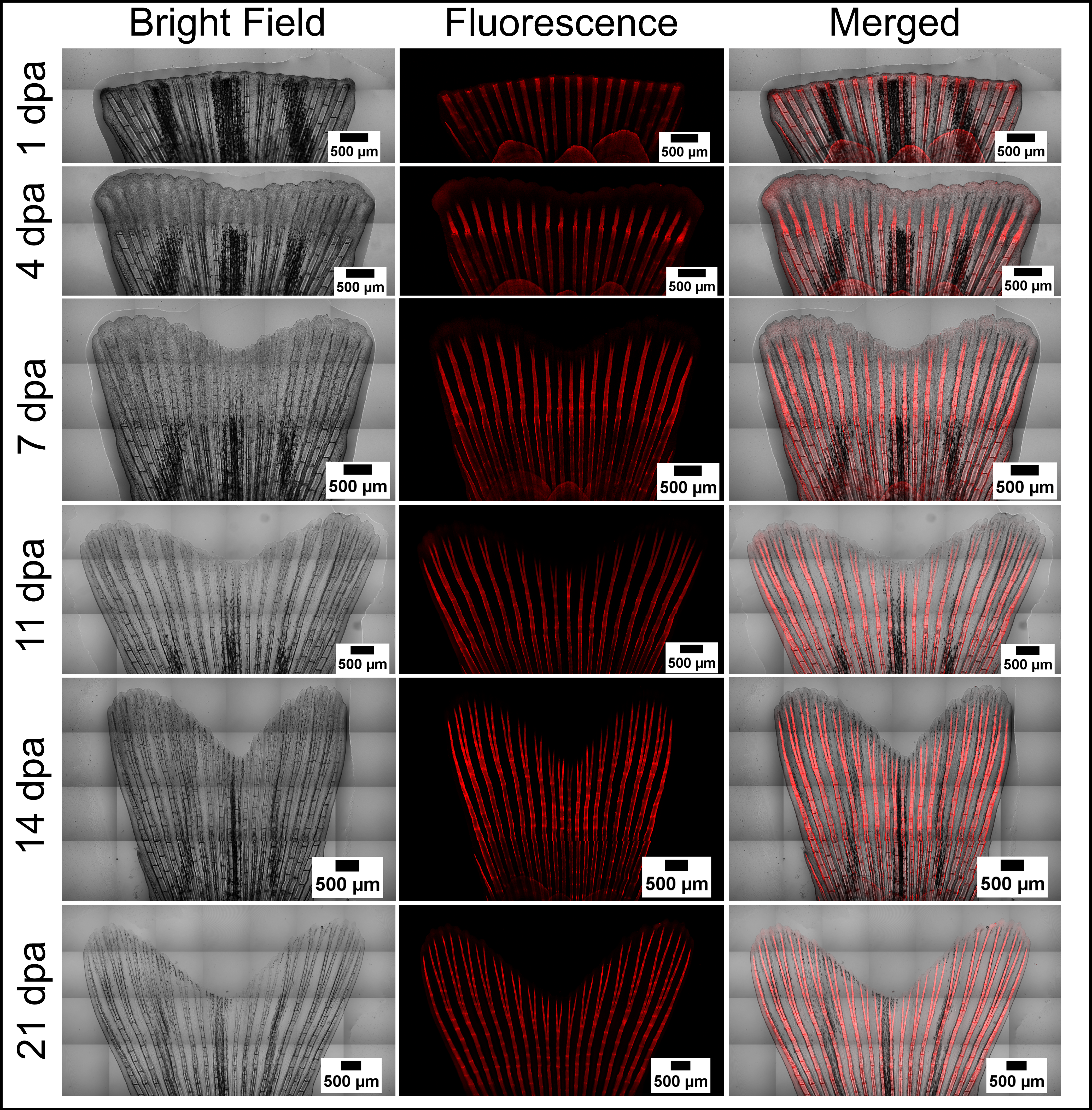

Supplement: Supplementary file 2 — Supplementary Fig. S2 Bone and tissue regeneration process of zebrafish caudal fin. The bright field column represents the tissue growth; the fluorescence column represents the de novo mineralization process; the merged column images represent the overall process (tissue + mineral) regeneration. The scale bar sizes are 500 μm. [file JBM4-5-e10435-s002.tif]
